# Editorial: Brain-inspired cognition and understanding for next-generation AI: Computational models, architectures and learning algorithms

**DOI:** 10.3389/fnins.2023.1169027

**Published:** 2023-03-24

**Authors:** Yuqi Han, Chenwei Deng, Guang-Bin Huang

**Affiliations:** ^1^School of Information and Electronics, Beijing Institute of Technology, Beijing, China; ^2^Department of Computer Science and Technology, Tsinghua University, Beijing, China; ^3^School of Electrical and Electronic Engineering, Nanyang Technological University, Singapore, Singapore

**Keywords:** perception science, brain-inspired cognition, artificial intelligence, multimodal perception, machine learning

## 1. Introduction

The human brain is probably the most complex thing in the universe. Apart from the human brain, no other system can automatically acquire new information and learn new skills, perform multimodal collaborative perception and information memory processing, make effective decisions in complex environments, and work stably with low power consumption. In this way, brain-inspired research can greatly advance the development of a new generation of artificial intelligence (AI) technologies.

Powered by new machine learning algorithms, effective large-scale labeled datasets, and superior computing power, AI programs have surpassed humans in speed and accuracy on certain tasks. However, most of the existing AI systems solve practical tasks from a computational perspective, eschewing most neuroscientific details, and tending to brute force optimization and large amounts of input data, making the implemented intelligent systems only suitable for solving specific types of problems. The long-term goal of brain-inspired intelligence research is to realize a general intelligent system. The main task is to integrate the understanding of multi-scale structure of the human brain and its information processing mechanisms, and build a cognitive brain computing model that attempt to simulate the cognitive function of the brain. In particular, attention needs to be paid to how the human brain cooperates with different computing components to accomplish different cognitive tasks such as perception, attention, learning, memorizing, knowledge representation, reasoning, decision-making, and judgment.

This special issue contains 14 research articles, which could be broadly classified into three classes: (1) three articles focus on investigating the spiking neural networks to explore the working mechanism for human brain, (2) three articles review several existing machine learning techniques and models by refering the working manner from human brain, (3) the remaining articles mainly focus on some practical applications such 3D modeling, robotics, speech recognition and image processing.

Specifically, Skatchkovsky et al. proposed a Bayesian learning framework for spiking neural networks (SNNs), which utilizes a Gaussian variational distribution for synaptic weights and a Bayesian single-task and continual learning rules with binary weights. Their study shows that the proposed framework has the ability to adapt to changing learning tasks and provides reliable quantification of uncertainty in the model's decisions. Hu and Liao proposed a membrane voltage slope-guided algorithm (VSG) that correlates delayed feedback signals with effective clues embedded in background spiking activity. This method finds potential points for emitting new spikes and the old spikes that need to be removed from the time derivative of membrane voltage, thereby avoiding the dilemma of failing to find adjustment points. Furthermore, it does not require iterative calculation to find the critical threshold. Zhao and Zeng proposed an intention prediction model for robots, which enables them to successfully predict user intentions through the spike-timing-dependent plasticity (STDP) mechanisms and simple feedback of right or wrong. Compared with the traditional Q-learning method, the proposed model significantly reduces training time.

Wingfield et al. proposed a deep artificial neural network model for speech processing that bears resemblance to patterns of activation in the human auditory cortex. This was achieved through a combination of spatio-temporal searchlight representational similarity analysis (ssRSA) and multimodal neuroimaging data. The study concludes that the low-dimensional bottleneck layer in the DNN could learn representations that characterize articulatory features of human speech. According to the study of Vaskevich and Torres, statistical learning is a highly dynamic and stochastic process that unfolds at different time scales, and evolves distinct learning strategies on demand. Their research reassesses how individuals dynamically learn predictive information in stable and unstable environments. Specifically, narrow-variance learners retain explicit knowledge of the regularity embedded in stimuli and use an error-correction strategy consistently in both stable and unstable environments. Broad-variance learners, on the other hand, emerge only in unstable environments. Lee et al. investigated brain-inspired predictive coding frameworks for machine challenging tasks (MCTs) and found that they have advantages in incremental learning, long-tailed recognition, and few-shot recognition. The study concludes that predictive coding learning is similar to the plasticity-stability property of the human brain, and mainly mimics the interaction between the hippocampus and prefrontal cortex.

For the practical application, Kumari et al. proposed an attentional search model for practical application in a 3D environment, utilizing two separate channels for object classification and location prediction. This enables the camera to accurately classify the target while focusing on it. Their model employs Elman and Jordan recurrence layers as well as JK-flip-flop recurrence layers instead of the traditional Long Short Term Memory (LSTM) to integrate temporal attention history into the network.

In the field of remote sensing, Shi et al. proposed an improved anchor-free SAR ship detection algorithm inspired by the brain's ability to effectively focus on target information and ignore interference from redundant information. The proposed model utilizes dense connection in the deep layer of the network and visual attention processing in the shallow layer to enhance feature extraction ability. Moreover, a wide height prediction constraint is applied to the target to further improve localization accuracy. Wang et al. proposed a knowledge-assisted neural network for millimeter wave radar object classification. This model injects two kinds of prior information containing spatial and physical understanding of objects for assistance. With the guidance of prior information, the network can learn object classification more akin to human brains and achieve superior performance. Tong et al. proposed an interpretable approach for automatic aesthetic assessment of remote sensing images. This method can highlight important areas of the image that influenced the model's decision, and provide visual explanations of the remote sensing aesthetic assessment.

Drawing inspiration from the way humans learn different object features based on the backgrounds and use historical appearances to aid in target positioning during tracking, Cui et al. proposed a novel tracking algorithm based on dynamic feature selection, aberrance repression, and a historical model retrieval module. By introducing dynamic feature-channel and aberrance repressed regularization into the loss function, the tracker can learn different feature weights according to the difference between the target and the background. The memory module, built by historical target samples, allows the tracker to learn a flexible representation that adapts to changes in object appearance during tracking. Similarily, Zheng et al. proposed a novel Gaussian prototype learning model to address tactile object recognition in open-set scenarios. Their unified framework integrates classification and class detection, consisting of two main components: a feature extractor and class prototypes. The feature extractor simulates the human perception system for transforming raw sensing data into abstract representations, and the prototypes for each category serve as abstract memories of the corresponding category in the brain. Experimental results validate that their model can not only correctly classify known tactile inputs but also effectively detect unknown tactile classes.

Current deep learning-based fundus image registration methods attempt to learn the geometric transformation or dense pixel-level displacement vector field directly between the test and reference images. However, the significant intra-class variability and small inter-class differences of fundus images pose a significant challenge for accurate keypoint matching. In response to this challenge, Xu et al. has proposed a spatially-varying adaptive pyramid context aggregation network to simultaneously match all the vessel crossing and branching points by taking advantage of the knowledge of contextual consistency.

Li et al. has proposed a kinship verification method based on face images that is relevant to real-life applications, such as missing children search, family photo classification, and kinship information mining. To enable the deep model to capture diverse and abundant local features from different regions of the face, they have proposed an attention center learning guided multi-head attention mechanism. To address the issue of misclassification caused by single feature center loss, a family-level multi-center loss has been proposed to ensure a more appropriate intra/inter-class distance measurement for kinship verification.

These articles cover a wide variety of topics including encoding and decoding of spatial-temporal information, 3-D environment modeling, visual object detection and localization, speech recognition, and aesthetic assessment. From the perspective of brain-inspired intelligence, these researches enrich the corresponding research fields with insightful methodologies and techniques, and ultimately offering alternative solutions to effectively enhance the robustness, generalization ability, and interpret ability for related tasks.

We hope that our readers will have a delightful experience when reading these excellent articles.

## Author contributions

YH prepared the orginal draft. CD and G-BH critically reviewed and edited the manuscript. All authors have reviewed and approved of the final manuscript.

